# Accessibility to Medication for Opioid Use Disorder After Interventions to Improve Prescribing Among Nonaddiction Clinics in the US Veterans Health Care System

**DOI:** 10.1001/jamanetworkopen.2021.37238

**Published:** 2021-12-06

**Authors:** Eric J. Hawkins, Carol A. Malte, Adam J. Gordon, Emily C. Williams, Hildi J. Hagedorn, Karen Drexler, Brittany E. Blanchard, Jennifer L. Burden, Jennifer Knoeppel, Anissa N. Danner, Aline Lott, Joseph G. Liberto, Andrew J. Saxon

**Affiliations:** 1 Seattle Center of Innovation for Veteran-Centered and Value-Driven Care, Health Services Research & Development, Veterans Affairs (VA) Puget Sound Health Care System, Seattle, Washington; 2 Center of Excellence in Substance Addiction Treatment and Education, VA Puget Sound Health Care System, Seattle, Washington; 3 Department of Psychiatry and Behavioral Sciences, University of Washington School of Medicine, Seattle; 4 Informatics, Decision-Enhancement, and Analytic Sciences Center, Health Services Research & Development, VA Salt Lake City Health Care System, Salt Lake City, Utah; 5 Program for Addiction Research, Clinical Care, Knowledge, and Advocacy, Department of Internal Medicine, University of Utah School of Medicine, Salt Lake City; 6 Department of Health Systems and Population Health, University of Washington, Seattle; 7 Center for Care Delivery & Outcomes Research, Health Services Research & Development, Minneapolis VA Health Care System, Minneapolis, Minnesota; 8 Department of Psychiatry, University of Minnesota, Minneapolis; 9 Office of Mental Health and Suicide Prevention, Veterans Health Administration, Washington, DC; 10 Department of Psychiatry and Behavioral Sciences, School of Medicine, Emory University, Atlanta, Georgia; 11 Department of Psychiatry, University of Maryland School of Medicine, Baltimore

## Abstract

**Importance:**

With increasing rates of opioid use disorder (OUD) and overdose deaths in the US, increased access to medications for OUD (MOUD) is paramount. Rigorous effectiveness evaluations of large-scale implementation initiatives using quasi-experimental designs are needed to inform expansion efforts.

**Objective:**

To evaluate a US Department of Veterans Affairs (VA) initiative to increase MOUD use in nonaddiction clinics.

**Design, Setting, and Participants:**

This quality improvement initiative used interrupted time series design to compare trends in MOUD receipt. Primary care, pain, and mental health clinics in the VA health care system (n = 35) located at 18 intervention facilities and nonintervention comparison clinics (n = 35) were matched on preimplementation MOUD prescribing trends, clinic size, and facility complexity. The cohort of patients with OUD who received care in intervention or comparison clinics in the year after September 1, 2018, were evaluated. The preimplementation period extended from September 1, 2017, through August 31, 2018, and the postimplementation period from September 1, 2018, through August 31, 2019.

**Exposures:**

The multifaceted implementation intervention included education, external facilitation, and quarterly reports.

**Main Outcomes and Measures:**

The main outcomes were the proportion of patients receiving MOUD and the number of patients per clinician prescribing MOUD. Segmented logistic regression evaluated monthly proportions of MOUD receipt 1 year before and after initiative launch, adjusting for demographic and clinical covariates. Poisson regression models examined yearly changes in clinician prescribing over the same time frame.

**Results:**

Overall, 7488 patients were seen in intervention clinics (mean [SD] age, 53.3 [14.2] years; 6858 [91.6%] male; 1476 [19.7%] Black, 417 [5.6%] Hispanic; 5162 [68.9%] White; 239 [3.2%] other race [including American Indian or Alaska Native, Asian, Native Hawaiian or other Pacific Islander, and multiple races]; and 194 [2.6%] unknown) and 7558 in comparison clinics (mean [SD] age, 53.4 [14.0] years; 6943 [91.9%] male; 1463 [19.4%] Black; 405 [5.4%] Hispanic; 5196 [68.9%] White; 244 [3.2%] other race; 250 [3.3%] unknown). During the preimplementation year, the proportion of patients receiving MOUD in intervention clinics increased monthly by 5.0% (adjusted odds ratio [AOR], 1.05; 95% CI, 1.03-1.07). Accounting for this preimplementation trend, the proportion of patients receiving MOUD increased monthly by an additional 2.3% (AOR, 1.02; 95% CI, 1.00-1.04) during the implementation year. Comparison clinics increased by 2.6% monthly before implementation (AOR, 1.03; 95% CI, 1.01-1.04), with no changes detected after implementation. Although preimplementation-year trends in monthly MOUD receipt were similar in intervention and comparison clinics, greater increases were seen in intervention clinics after implementation (AOR, 1.04; 95% CI, 1.01-1.08). Patients treated with MOUD per clinician in intervention clinics saw greater increases from before to after implementation compared with comparison clinics (incidence rate ratio, 1.50; 95% CI, 1.28-1.77).

**Conclusions and Relevance:**

A multifaceted implementation initiative in nonaddiction clinics was associated with increased MOUD prescribing. Findings suggest that engagement of clinicians in general clinical settings may increase MOUD access.

## Key Points

**Question:**

Was a multifaceted implementation intervention associated with increased access to medications for opioid use disorder (MOUD) in clinics not specializing in addiction treatment?

**Findings:**

A quality improvement implementation initiative across 35 nonaddiction clinics (primary care, pain, and mental health) seeing a total of 7488 patients in 18 Veterans Affairs facilities was associated with increased prescribing of specific MOUD.

**Meaning:**

These results suggest that a multifaceted implementation initiative that engages clinicians in general clinical settings may increase access to MOUD.

## Teaser

This quality improvement study assesses whether a Veterans Affairs initiative was associated with increased prescribing of medications for opioid use disorder.

## Introduction

Opioid-related deaths are increasing, and untreated opioid use disorder (OUD) remains common in the US.^1,2,3,4,5^ In 2019, more than 50 000 individuals died of opioid-related overdose, and 2 million individuals were estimated to have an OUD.^6^ Opioid-related deaths and fentanyl use have been increasing during the coronavirus pandemic.^7,8^ Although medication treatment for OUD (MOUD), which includes methadone, buprenorphine, and naltrexone, reduces opioid use, overdose, and mortality,^9,10,11,12,13,14^ it is underused.^15,16^

To address an estimated 1 million individuals with untreated OUD, federal and state governments have funded initiatives to improve access to MOUD.^17,18,19,20,21,22^ However, few evaluations have assessed the effectiveness of these initiatives.^23^ Initiatives in California, Washington, and West Virginia focused on increasing MOUD access through implementing a hub-and-spoke model, which includes a hub, typically a substance use disorder (SUD) specialty clinic, and a network of clinicians and health care settings (spokes), often primary care clinics.^17,24,25,26^ Preliminary results indicated increases in prescribers obtaining US Drug Enforcement Administration waivers and buprenorphine initiation.^23,25,27,28^ Although results are promising, most studies^23,27,28,29^have used pre-post, single-arm designs and/or focused on SUD specialty care.

Despite several US Department of Veteran Affairs (VA) initiatives to increase access to MOUD, in 2017 a total of 35% of patients with an OUD in the VA health care system received MOUD, largely from SUD specialty care.^2^ To enhance MOUD access, in 2018 the VA developed and implemented the Stepped Care for Opioid Use Disorder Train-the-Trainer (SCOUTT) initiative.^30,31^ SCOUTT aimed to improve access to MOUD, specifically buprenorphine and injectable naltrexone, in primary care, pain, and mental health clinics, using a multifaceted implementation intervention.^30^ Stepped care, a population-based approach to screening, assessment, and management of OUD, prioritizes matching patient needs to the least resource-intensive interventions in general health care settings first. Those with more complex needs are stepped up to SUD specialty care as needed. Stepped-care models have been described for several conditions, including addiction, depression, anxiety, and pain.^32,33,34,35,36^

The current project evaluated the SCOUTT initiative’s expansion of MOUD to primary care, pain, and mental health clinics (hereafter referred to as intervention clinics). Using an interrupted time series design,^37^ we compared intervention clinics and matched comparison clinics not receiving the SCOUTT intervention on the proportion of patients with OUD receiving MOUD, proportion of clinicians prescribing MOUD, and patients treated per clinician.

## Methods

### Setting

The setting included intervention clinics (n = 35) in 18 VA facilities, 1 from each of the VA’s 18 regional networks. Comparison clinics (n = 35) were selected based on similarity to intervention clinics with respect to baseline-level MOUD prescribing trends, clinic size (number of patients with OUD), and facility-level complexity (eMethods and eTable 1 in the Supplement).^38^

### Evaluation Design

This evaluation used an interrupted time series design to evaluate trend and level changes in MOUD receipt among patients with OUD during the year before and year after the intervention. SCOUTT launched on September 1, 2018. Given the VA’s priority to increase MOUD and several smaller VA initiatives that target this goal after the launch of SCOUTT, analyses were limited to 1 year after launch; thus, the preimplementation period extended from September 1, 2017, through August 31, 2018, and the postimplementation period from September 1, 2018, through August 31, 2019. To address potential influences of other national VA MOUD initiatives,^2,39^ we compared MOUD-prescribing trends in intervention clinics with those of matched clinics at VA facilities with no participation in the SCOUTT initiative. A unique patient identifier generated by the VA Corporate Data Warehouse was retained. No other patient identifiers were maintained. This evaluation was classified as quality improvement by the VA Office of Mental Health and Suicide Prevention and did not require institutional review board approval; the study followed the Standards for Quality Improvement Reporting Excellence (SQUIRE) reporting guideline.^40^

### Procedures

**Figure 1.**
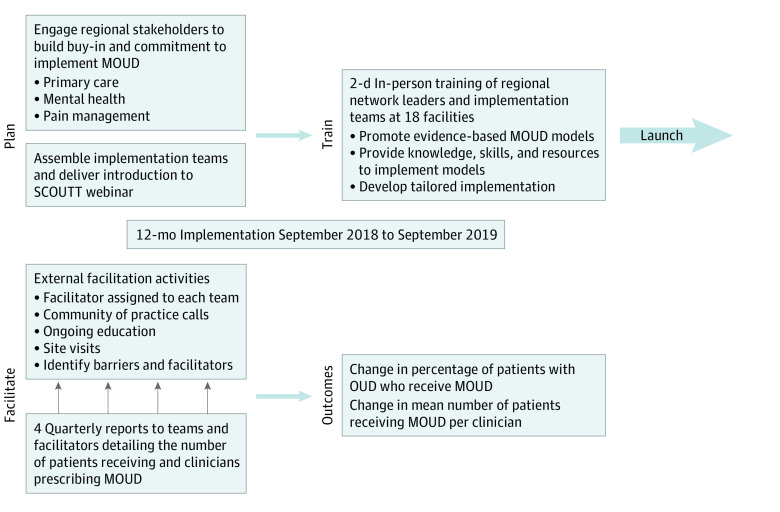
Stepped Care for Opioid Use Disorder Train-the-Trainer (SCOUTT) Initiative Overview MOUD indicates medications for opioid use disorder; OUD, opioid use disorder.

Figure 1 provides a SCOUTT overview. On May 1, 2018, a memorandum was sent to 18 VA regional network directors, introducing the SCOUTT initiative and requesting they identify a network facility to participate and assemble a cross-disciplinary implementation team.^41,42,43^ Teams consisted of 4 to 5 clinicians, including prescribers, nurses, behavioral specialists, and pharmacists, from at least 1 intervention clinic.^30^ Each facility also assembled an SUD care team to consult with implementation teams. Networks had full autonomy to select facilities within their networks.

### Intervention

The intervention was guided by the integrated Promoting Action on Research Implementation in Health Services framework, which proposes successful implementation as a function of 4 interacting elements: ability to innovate an intervention into settings, influence of recipients involved in implementation, quality of implementation context, and facilitation of implementation process.^44^ The multifaceted intervention approach included interventions known to promote these elements (eTable 2 in the Supplement).^45^ SCOUTT details have been previously reported.^30,46^ Training before launch included 2 webinars, followed by an in-person 2-day meeting in August 2018. Trainings introduced SCOUTT and 2 evidenced-based MOUD models: physician-led medical management and nurse-led collaborative care.^41,42,43^ Training included provision of knowledge and resources to facilitate implementation of delivery models and guided activities to develop implementation strategies tailored to intervention clinics. After training, teams were tasked with increasing access to MOUD in their clinic(s). SCOUTT implementation used ongoing external facilitation, including meetings with implementation teams to identify and address barriers, monthly virtual meetings to promote community building, webinars to address knowledge gaps, and expert consultation and/or site visits. Implementation teams received quarterly reports of monthly numbers of clinicians prescribing MOUD, numbers of patients receiving MOUD, and percentages of patients receiving MOUD for 90 days or more in intervention clinics. Implementation teams were encouraged but not required to use any of the specific interventions. Implementation teams’ use of these interventions is detailed in eTable 2 in the Supplement.

In-person training costs were reimbursed, but facilities received no additional financial support. Evaluation and facilitation activities were funded by the VA Quality Enhancement Research Initiative.^47,48^

### Data Sources

This evaluation used the VA Corporate Data Warehouse, a data repository of patient-level data from VA electronic medical records. Diagnostic and encounter data were used to identify patients with OUD in intervention and comparison clinics. Orders, VA pharmacy files, fee basis pharmacy files, and non-VA pharmacy files were linked with encounter data to provide information on medication(s) prescribed, days’ supply, and prescription sources (clinician and clinic). Patient and clinician demographic information was also extracted from the VA Corporate Data Warehouse.

### Cohort of Patients With OUD

Patients 18 years or older with documented OUD during 12 months before or after SCOUTT launch and 1 or more outpatient visits in intervention or comparison clinics during 12 months after launch were included in the analyses.

### Outcome Measures

The primary outcome was the monthly proportion of patients who were prescribed MOUD (buprenorphine or injectable naltrexone) in intervention and comparison clinics during 12 months before and after SCOUTT launch. Prescription data identified all MOUD prescriptions for included patients. Clinic codes associated with prescriptions were used to determine the clinic where prescriptions originated. A binary indicator (yes/no) was used to identify MOUD prescriptions from intervention and comparison clinics for each of the 12 months before and after implementation for every patient. Changes in MOUD trends in intervention clinics were compared with those in comparison clinics.

Secondary outcomes included clinician activity 12 months before and after SCOUTT launch: changes in the proportion of clinicians prescribing MOUD to patients with OUD and changes in the number of patients prescribed MOUD per clinician among clinicians who prescribed MOUD during the implementation year. Measures of retention, defined as the proportion of patients receiving 90 days or more and 180 days or more of MOUD, without a break in medication supply exceeding 30 days, were calculated for intervention and comparison clinics. Time receiving MOUD was calculated from date of first medication prescription filled in intervention and comparison clinics. Subsequent prescriptions from nonintervention clinics were included to allow for transfers in care.

We included patient-specific covariates that may contribute to differences in MOUD receipt in models that evaluated the primary outcome. Demographic covariates included age, sex, race, ethnicity, marital status, housing insecurity, and VA disability rating of 50% or greater (ie, an injury or illness from military service that determines VA health care eligibility and benefits). Race and ethnicity data were extracted from the VA Corporate Data Warehouse to characterize the sample. Clinical covariates included mental health (depression, anxiety, posttraumatic stress disorder, and bipolar and psychotic disorders), SUD (alcohol use disorder and any non–opioid drug use disorder), and medical comorbidity, based on *International Statistical Classification of Diseases, Tenth Revision, Clinical Modification (ICD-10-CM) *codes from VA outpatient and inpatient care. The Charlson Comorbidity Index^38^ measured medical comorbidity.^39^ Clinic type (primary care, mental health, or pain) and an indicator for each of the 18 intervention facilities were included to account for practice differences. Three monthly, time-varying binary indicators were calculated: visit attendance in intervention and comparison clinics, SUD specialty care attendance, and MOUD receipt from other clinics, including VA and non-VA clinics and opioid treatment programs. Clinician-specific covariates for analyses included prescriber discipline (physician, physician resident, nurse practitioner, and physician assistant), number of patients with OUD seen during implementation year, VA facility-level complexity, and clinic type. Clinical characteristics were from electronic medical record data 1 year before SCOUTT launch unless specified.

### Statistical Analysis

Descriptive statistics were used to characterize patients with OUD in the intervention and comparison clinics. Change in prescribing trends during the year before and after SCOUTT launch was evaluated using segmented logistic regression with interrupted time series.^37^ Models used patients as the analysis unit and estimated change over time in the proportion of patients receiving MOUD in intervention clinics after launch, adjusting for patient-specific covariates detailed above, clinic type, time-varying indicators of treatment receipt, and prescribing trends 12 months before launch. Models included terms for outcome (MOUD prescribed in intervention and comparison clinics; 0 = comparison, 1 = intervention) for each of 24 months, month since beginning of prelaunch period (1-24) to estimate secular trends, month since initiative launch (13-24) to estimate change in trend after launch, and a pre/postinitiative launch indicator (0 = prelaunch [months 1-12], 1 = postlaunch [months 13-24]) to estimate immediate change after launch. Given the hierarchical data structure, mixed-effects logit models were run with both patient and site added as random effects, but the models failed to converge. To account for correlation at the patient level, segmented logistic regressions used generalized estimating equations (xtgee) with a logit link, an autoregressive (AR1) correlation structure, and site included as the fixed effect. Changes in trend and level (immediate change) between the intervention and comparison clinics were compared using similarly adjusted models with an interaction term for clinic (intervention vs comparison). In sensitivity analyses, we ran mixed-effects logit models that include site only as a random effect, adjusting for patient-specific covariates. Mixed-effects logit models estimated differences between the intervention and comparison clinics in change in proportion of clinicians prescribing MOUD in the year before and after SCOUTT launch, adjusting for clinician-specific covariates and including site and clinician as random effects. Mixed-effects Poisson models compared clinics in pre/postinitiative change in the number of patients prescribed MOUD per clinician among clinicians with 1 or more MOUD prescription during the implementation year, adjusting for covariates and including site and clinician as random effects. The proportion of patients receiving 90 days or more and 180 days or more of a continuous medication supply in intervention and comparison clinics was analyzed using logistic regression, adjusted for patient-specific covariates.

Analyses were conducted using Stata MP, version 15.1 (StataCorp LLC). A 1-sided *P* < .05 was considered to be statistically significant.

## Results

Overall, 7488 patients were seen in intervention clinics (mean [SD] age, 53.3 [14.2] years; 6858 [91.6%] male; 1476 [19.7%] Black, 417 [5.6%] Hispanic; 5162 [68.9%] White; 239 [3.2%] other race [including American Indian or Alaska Native, Asian, Native Hawaiian or other Pacific Islander, and multiple races]; and 194 [2.6%] unknown) and 7558 in comparison clinics (mean [SD] age, 53.4 [14.0] years; 6943 [91.9%] male; 1463 [19.4%] Black; 405 [5.4%] Hispanic; 5196 [68.9%] White; 244 [3.2%] other race; 250 [3.3%] unknown) (Table 1). In the intervention group, 4103 patients (54.8%) had 50% or greater service disability, and 1800 (24.0%) had insecure housing. The most common comorbid substance use and mental health conditions were alcohol use (2586 [34.5%]) and depressive disorders (3354 [44.8%]). A total of 2432 patients (32.5%) received MOUD and 2226 (29.7%) attended SUD specialty care during the preimplementation year. Compared with the intervention clinics, comparison clinic patients (n = 7558) were less likely diagnosed with posttraumatic stress disorder (2975 [39.4%] vs 3267 [43.6%]) or to have received MOUD in the prior year (1894 [26.4%] vs 2432 [32.5%]). Unstable housing (1605 [26.3%] vs 1800 [24.0%]), alcohol use (2895 [38.3%] vs 2586 [34.5%]), anxiety disorders (2704 [35.8%] vs 2426 [32.4%]), and prior year SUD specialty care attendance (2596 [34.4%] vs 2226 [29.7%]) were more common among comparison clinic patients. The most common intervention clinic was primary care (n = 17) followed by pain (n = 11) and mental health (n = 7).

**Table 1.** Baseline Patient Characteristics at Intervention and Comparison Clinics

Characteristic		Comparison clinics (n = 7558)	Intervention clinics (n = 7488)	Standardized mean difference
Age, mean (SD), y		53.4 (14.0)	53.3 (14.2)	−0.4
Sex				
Male		6943 (91.9)	6858 (91.6)	−1.0
Female		615 (8.1)	630 (8.4)	1.0
Marital status				
Married		2282 (30.2)	2432 (32.5)	4.9
Not married		5256 (69.5)	5025 (67.1)	−5.2
Unknown		20 (0.3)	31 (0.4)	2.6
Service disability ≥50%		3976 (52.6)	4103 (54.8)	4.4
Race and ethnicity				
Black		1463 (19.4)	1476 (19.7)	0.9
Hispanic		405 (5.4)	417 (5.6)	0.9
White		5196 (68.9)	5162 (68.9)	0.4
Other race^a^		244 (3.2)	239 (3.2)	−0.2
Unknown		250 (3.3)	194 (2.6)	−4.2
Unstable housing		1605 (26.3)	1800 (24.0)	−6.9
Substance use disorder diagnoses				
Alcohol use		2895 (38.3)	2586 (34.5)	−7.8
Cannabis use		1443 (20.1)	1398 (18.7)	− 3.6
Stimulant use		2062 (28.7)	1996 (26.7)	−4.6
Any nonopioid drug use		3113 (41.2)	2951 (39.4)	−3.6
Mental health disorder diagnoses				
Serious mental illness^b^		1425 (18.8)	1309 (17.5)	−3.6
Depressive		3358 (44.4)	3354 (44.8)	0.7
Anxiety		2704 (35.8)	2426 (32.4)	−7.1
Posttraumatic stress disorder		2975 (39.4)	3267 (43.6)	8.7
No. of mental health diagnoses				
0		1878 (24.9)	1858 (24.8)	−0.1
1		2134 (28.2)	2141 (28.6)	0.8
≥2		3546 (46.9)	3489 (46.6)	−0.6
Charlson Comorbidity Index				
0		3548 (46.9)	3789 (50.6)	7.3
1		1731 (22.9)	1550 (20.7)	−5.3
≥2		2279 (30.2)	2149 (28.7)	−3.2
Prior year				
MOUD receipt		1894 (26.4)	2432 (32.5)	13.5
SUD specialty care visits		2596 (34.4)	2226 (29.7)	−9.9
Clinic type				
Mental health		2638 (34.9)	2293 (30.6)	−9.1
Primary care		3613 (47.8)	4172 (55.7)	−1.01
Pain	1307 (17.3)		1023 (13.7)	15.9

Abbreviations: MOUD, medications for opioid use disorder (defined as buprenorphine, injectable naltrexone, and methadone); SUD, substance use disorder.

^a^ Other race includes American Indian or Alaska Native, Asian, Native Hawaiian or other Pacific Islander, and multiple races.

^b^ Serious mental illness disorders include schizophrenia-spectrum or bipolar-spectrum disorders.

### Patients Prescribed MOUD

**Figure 2.**
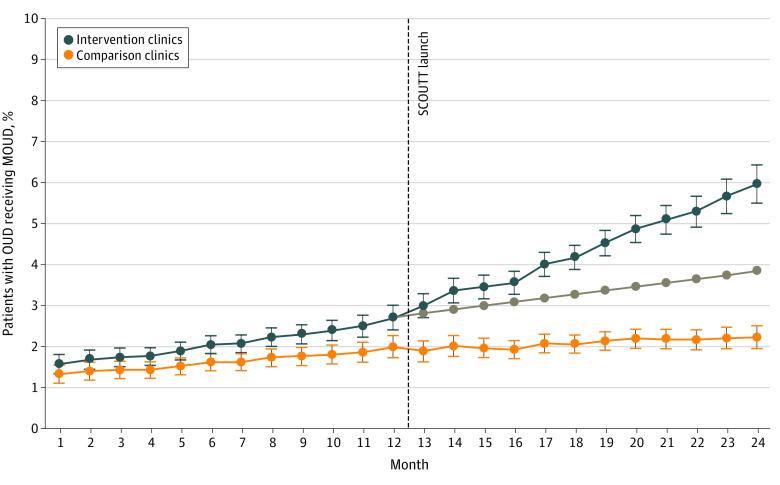
Adjusted Percentage of Patients Receiving Medications for Opioid Use Disorder in Intervention and Comparison Clinics Error bars indicate 95% CI; gray line, preimplementation trend. MOUD indicates medications for opioid use disorder; OUD, opioid use disorder; SCOUTT, Stepped Care for Opioid Use Disorder Train-the-Trainer.

Among intervention clinics, the proportion of patients receiving MOUD increased 5.0% (adjusted odds ratio [AOR], 1.05; 95% CI, 1.03-1.07; *P* < .001) monthly during the preimplementation period (from 1.6% in month 1 to 2.7% in month 12) (Figure 2). During the implementation period, the proportion receiving MOUD increased an additional 2.3% (AOR, 1.02; 95% CI, 1.00-1.04; *P* = .04) monthly over the secular trend (to 6.0% in month 24), reflecting an absolute increase of 3.3% and relative increase of 122.2%. No change in MOUD receipt occurred immediately after SCOUTT launch (AOR, 1.01; 95% CI, 0.91-1.12). Figure 2 shows trends in intervention clinics compared with trends in comparison clinics.

Comparison clinics saw increases in preimplementation trends (AOR, 1.03; 95% CI, 1.01-1.04; *P* = .001). No additional postimplementation increase was detected (AOR, 0.99; 95% CI, 0.97-1.01). A decrease in MOUD receipt occurred immediately after launch (AOR, 0.88; 95% CI, 0.78-0.99; *P* = .03). The percentage of patients receiving MOUD increased from 1.3% at month 1 to 2.0% at month 12 before implementation and to 2.2% at month 24, reflecting an absolute increase of 0.2% and relative increase of 10.0% in the implementation year. Comparing intervention and comparison clinic trends, no differences were detected in preimplementation secular trends (AOR, 1.01; 95% CI, 0.99-1.04); implementation clinics had greater increases in MOUD after implementation (AOR, 1.04; 95% CI, 1.01-1.08; *P* = .006). The AOR for step change for intervention vs comparison clinics was 1.13 (95% CI, 0.97-1.32; *P* = .12).

In sensitivity analyses, changes in trends and levels for intervention and comparison clinics were similar, as were differences between clinics (eTable 3 in the Supplement).

Because monthly estimates do not show the cumulative rate of MOUD receipt, we also report annual rates of MOUD receipt. From pre- to postimplementation years, the unadjusted annual percentage of MOUD receipt increased from 6.3% (95% CI, 5.8%-6.9%) to 13.6% (95% CI, 12.8%-14.4%) in intervention clinics (eTable 4 in the Supplement). In comparison clinics, MOUD receipt was 4.2% (95% CI, 3.8%-4.7%) and 6.1% (95% CI, 5.6%-6.7%) in pre/postimplementation years, respectively. Most MOUD prescriptions in the intervention and comparison clinics during the implementation year were for buprenorphine (92.9% for intervention clinics and 89.2% for comparison clinics).

### Clinicians Prescribing MOUD

From the pre/postimplementation years, the adjusted percentage of clinicians prescribing MOUD increased from 8.6% (95% CI, 6.8%-10.4%) to 13.9% (95% CI, 11.6%-16.1%) in intervention clinics (*P* < .001) and from 6.1% (95% CI, 4.0%-8.2%) to 8.2% (95% CI, 5.7%-10.6%) in comparison clinics (*P* = .006), with no difference in percent change detected among clinics. Among clinicians with 1 or more MOUD prescription during the implementation year, the mean number of patients receiving MOUD per clinician increased by an estimated 7.40 (95% CI, 3.91-10.89; incidence rate ratio [IRR], 2.41; 95% CI, 2.20-2.63) patients in intervention clinics and 2.32 (95% CI, 1.33-3.31; IRR, 1.60; 95% CI, 1.40-1.83) patients in comparison clinics from preimplementation to implementation year, adjusting for covariates (Table 2). Per clinician, greater increases in patients treated with MOUD per year were seen in intervention compared with comparison clinics (IRR, 1.50; 95% CI, 1.28-1.77, *P* < .001).

**Table 2.** Mean Number of Patients Receiving MOUD Among Clinicians in Comparison and Intervention Clinics

Clinic	Patients per clinician			IRR (95% CI)^a^
	Preimplementation year	Postimplementation year		
Comparison clinics	3.87 (2.58- 5.16)	6.19 (4.17-8.22)		1.60 (1.40-1.83)^b^
Intervention clinics	5.25 (2.80-7.71)	12.65 (6.78-18.53)	2.41 (2.20-2.63)^b^	

Abbreviations: IRR, incidence rate ratio; MOUD, medications for opioid use disorder.

^a^ Adjusted for number of unique patients with opioid use disorder seen in implementation year, clinic type (primary care, mental health, or pain), clinician discipline, and US Department of Veterans Affairs facility complexity. Site and clinician included as random effects.

^b^ *P* < .001.

### Retention of Patients

Among 987 patients who received MOUD in intervention clinics during the implementation year, an estimated 70.3% (95% CI, 67.6%-73.1%) continued to receive medication for 90 days or more and 60.2% (95% CI, 57.2%-63.1%) for 180 days or more in their first treatment episode. In comparison clinics, among 441 patients prescribed MOUD, 82.1% (95% CI, 78.6%-85.7%) were retained for 90 days or more and 68.7% (95% CI, 64.4%-73.0%) for 180 days or more. Intervention clinic patients were less likely retained for 90 days or more (AOR, 0.48; 95% CI, 0.35-0.65; *P* < .001) and 180 days or more (AOR, 0.66; 95% CI, 0.51-.86; *P* = .002).

## Discussion

In this quality improvement evaluation of a multicomponent implementation intervention to improve MOUD delivery, there was an associated increase in MOUD-prescribing among patients in intervention clinics, with intervention clinics seeing greater increases in MOUD-prescribing compared with comparison clinics after implementation. During the initial year, monthly rates of MOUD increased by 3.3% in absolute terms and 122.2% in relative terms. We found a larger increase in the number of patients receiving MOUD per clinician at intervention clinics compared with comparison clinics, further supporting the intervention’s influence. However, patients in intervention clinics were less likely to be retained for more than 90 or 180 days than patients in comparison clinics. Several studies^23,25,43^ have reported improvements in prescribing outcomes after initiating strategies to increase access to MOUD using hub-and-spoke and nurse care manager models, and several rigorously designed studies are underway,^49^ but to our knowledge no prior evaluation has adjusted for secular trends or compared implementation sites to similar sites without intervention exposure.

Although these findings suggest that the multistrategy implementation intervention was effective in increasing MOUD delivery at intervention clinics, whether all or any of the specific intervention strategies are associated with prescribing increases is unknown. To inform efforts to expand MOUD across the US, determining which specific intervention strategies are associated with increased access, the extent to which a team approach facilitated implementation, and if any strategies were underused is critical. Identifying influential intervention strategies can inform changes to intervention approaches and address the interests of other health care systems planning to increase MOUD access. Given the diversity of intervention clinics in this evaluation, it will be important to examine differences in implementation across primary care, mental health, and pain clinics and determine whether specific implementation strategies were more effective for certain clinic types.

Our finding that retention was lower among intervention clinics may be attributable to several factors. Differences in patient motivation and/or readiness for care may account for this finding because efforts to expand access to treatment may include patients less able to be motivated to change their behavior.^50,51^ The quality of clinician-patient interactions, particularly among less experienced clinicians, may play a role as well. Regardless, it will be important to monitor retention over time and identify factors that may account for lower retention observed among intervention clinics.

### Limitations

This study has limitations. Although we took steps to address secular trends, adjusted for demographic and clinical covariates, and compared trends with comparison clinics without SCOUTT involvement, results may be influenced by residual confounding or several other ongoing VA initiatives to increase MOUD. The prescribing trends presented are limited to the initial implementation year and do not address the sustainability of MOUD delivery, an implementation outcome of importance to health care systems.^52^ Furthermore, our sample consisted of VA patients, and results may not generalize to nonveterans or veterans not receiving VA care. In addition, our estimates of MOUD receipt underestimate MOUD delivery in the VA, currently greater than 44% for patients with OUD, because prescriptions were limited to clinicians in clinics under evaluation and do not include those from SUD specialty care clinics, where most MOUD are delivered.

## Conclusions

Taken together, the results of this evaluation suggest that a multifaceted implementation approach used to engage clinicians in primary care, mental health, and pain clinics in MOUD delivery is associated with an increase in prescriber productivity and patient access to MOUD. Implementing SCOUTT-like initiatives in clinics with infrastructure to support MOUD prescribing may assist health care systems in reaching patients who do not access traditional OUD treatment. Future investigations will determine whether specific implementation strategies are more effective for certain clinic types and patient populations.

## Notes

Supplement. **eMethods.** Approach Used to Match Comparison Clinics to Intervention Clinics **eTable 1.** Characteristics of Comparison and Intervention Clinics During Preimplementation Year **eTable 2.** Integrated Promoting Action on Research Implementation in Health Services Framework Elements and SCOUTT Initiative Interventions **eTable 3.** Sensitivity Analyses—Association Between the Implementation Intervention and Monthly Proportion of Patients Receiving MOUD Using Mixed Effects Logit Models With Clinic Only as Random Effect **eTable 4.** Yearly Unadjusted Percentages of MOUD Receipt Among Patients in Comparison and Intervention Clinics
