# Supplementary material for: Accessibility to Medication for Opioid Use Disorder After Interventions to Improve Prescribing Among Nonaddiction Clinics in the US Veterans Health Care System
Source: JAMA Netw Open. 2021 Dec 6;4(12):e2137238. doi: 10.1001/jamanetworkopen.2021.37238 (PMC8649831; doi:10.1001/jamanetworkopen.2021.37238)
Supplement: Supplement. — eMethods. Approach Used to Match Comparison Clinics to Intervention Clinics eTable 1. Characteristics of Comparison and Intervention Clinics During Preimplementation Year eTable 2. Integrated Promoting Action on Research Implementation in Health Services Framework Elements and SCOUTT Initiative Interventions eTable 3. Sensitivity Analyses—Association Between the Implementation Intervention and Monthly Proportion of Patients Receiving MOUD Using Mixed Effects Logit Models With Clinic Only as Random Effect eTable 4. Yearly Unadjusted Percentages of MOUD Receipt Among Patients in Comparison and Intervention Clinics [file jamanetwopen-e2137238-s001.pdf]

# Supplementary Online Content

Hawkins EJ, Malte CA, Gordon AJ, et al. Accessibility to medication for opioid use disorder after interventions to improve prescribing among nonaddiction clinics in the US veterans health care system. *JAMA Netw Open*. 2021;4(12):e2137238. doi:10.1001/jamanetworkopen.2021.37238

**eMethods.** Approach Used to Match Comparison Clinics to Intervention Clinics

**eTable 1.** Characteristics of Comparison and Intervention Clinics During Preimplementation Year

**eTable 2.** Integrated Promoting Action on Research Implementation in Health Services Framework Elements and SCOUTT Initiative Interventions

**eTable 3.** Sensitivity Analyses—Association Between the Implementation Intervention and Monthly Proportion of Patients Receiving MOUD Using Mixed Effect Logit Models With Clinic Only as Random Effect

**eTable 4.** Yearly Unadjusted Percentages of MOUD Receipt Among Patients in Comparison and Intervention Clinics

**This supplementary material has been provided by the authors to give readers additional information about their work.**

© 2021 Hawkins EJ et al. *JAMA Network Open.*

## eMethods. Approach Used to Match Comparison Clinics to Intervention Clinics

To increase similarity between intervention and comparison clinics with respect to secular trends in MOUD prescribing, intervention clinics were matched 1:1 to comparison clinics using nearest-neighbor matching in Stata 15 (teffects nnmatch). Intervention clinics, identified both by clinic location and type (mental health, primary care, pain management; n=35), and comparison clinics, which included all mental health, primary care, pain management that treated patients with OUD in the preimplementation year, were entered as treatment variables (1=intervention clinics, 0=comparison clinics). Variables in the models included clinic-specific count of patients with OUD treated in the preimplementation year; clinic-specific percent of patients receiving MOUD by month in the preimplementation year; and facility-level complexity. A unique comparison clinic was selected for each intervention clinic, for a total of 70 clinics.

© 2021 Hawkins EJ et al. *JAMA Network Open.*

## eTable 1. Characteristics of Comparison and Intervention Clinics in the Pre-implementation Year

| | | Pre-implementation Comparison Clinics (n=35) | | | | | | | Pre-implementation Intervention Clinics (n=35) | | | | | |
|---|---|---|---|---|---|---|---|---|---|---|---|---|---|---|
| **Facility** | **Clinic Type** | **Facility Complexity[a]** | **Prescriber[b] No.*** | **MDs[c] %** | **Patients with OUD[d] No.** | **Patients Receiving MOUD[e] %** | **Increase MOUD Receipt[f] %** | | **Facility Complexity[a]** | **Prescriber[b] No.*** | **MDs[c] %** | **Patients with OUD[d] No.** | **Patients Receiving MOUD[e] %** | **Increase MOUD Receipt[f] %** |
| 1 | MH | High | ≥50 | 83.9 | ≥500 | >5% | <1% | | High | ≥50 | 90.0 | ≥500 | >5% | <1% |
| | PC | High | ≥50 | 84.6 | ≥500 | 1 - 5% | <1% | | High | ≥50 | 63.6 | ≥500 | >5% | 1 - 5% |
| 2 | PC | High | ≥50 | 57.1 | 100-499 | 1 - 5% | <1% | | High | 10-24 | 95.2 | 100-499 | <1% | <1% |
| 3 | PC | High | ≥50 | 66.7 | 100-499 | <1% | <1% | | High | ≥50 | 92.2 | ≥500 | <1% | <1% |
| | Pain | High | 10-24 | 68.8 | <100 | >5% | 1 - 5% | | High | <10 | 66.7 | <100 | 1 - 5% | <1% |
| 4 | MH | High | 25-49 | 58.6 | 100-499 | >5% | 1 - 5% | | High | <10 | 100.0 | <100 | >5% | 1 - 5% |
| | PC | High | 10-24 | 93.8 | 100-499 | <1% | <1% | | High | 10-24 | 77.3 | 100-499 | >5% | <1% |
| | MH | High | ≥50 | 69.4 | ≥500 | >5% | <1% | | High | 25-49 | 84.1 | ≥500 | >5% | 1 - 5% |
| | Pain | High | 10-24 | 81.8 | <100 | 1 - 5% | 1 - 5% | | High | 10-24 | 72.7 | 100-499 | >5% | >5% |
| 5 | PC | High | ≥50 | 74.6 | ≥500 | <1% | <1% | | High | ≥50 | 78.6 | ≥500 | 1 - 5% | <1% |
| 6 | PC | Low | 25-49 | 65.4 | 100-499 | <1% | <1% | | Low | <10 | 100.0 | <100 | <1% | <1% |
| | PC | High | 25-49 | 75.0 | 100-499 | 1 - 5% | <1% | | Low | 10-24 | 78.6 | 100-499 | <1% | <1% |
| 7 | PC | High | <10 | 0.0 | <100 | <1% | <1% | | High | <10 | 60.0 | <100 | <1% | <1% |
| | Pain | High | <10 | 42.9 | 100-499 | <1% | <1% | | High | <10 | 87.5 | <100 | <1% | <1% |
| 8 | Pain | High | <10 | 83.3 | 100-499 | 1 - 5% | 1 - 5% | | High | 10-24 | 60.0 | 100-499 | >5% | >5% |
| 9 | Pain | High | <10 | 87.5 | 100-499 | <1% | <1% | | High | <10 | 55.6 | <100 | <1% | <1% |
| 10 | PC | High | ≥50 | 67.3 | 100-499 | 1 - 5% | <1% | | High | 10-24 | 100.0 | 100-499 | 1 - 5% | <1% |
| | Pain | High | <10 | 100.0 | <100 | >5% | >5% | | High | <10 | 100.0 | <100 | >5% | >5% |
| 11 | MH | High | ≥50 | 100.0 | 100-499 | >5% | 1 - 5% | | High | 25-49 | 69.2 | 100-499 | 1 - 5% | <1% |

© 2021 Hawkins EJ et al. *JAMA Network Open.*

| | | | | | | | | | | | | | | |
|---|---|---|---|---|---|---|---|---|---|---|---|---|---|---|
| | MH | High | 25-49 | 69.4 | 100-499 | 1 - 5% | <1% | | High | 25-49 | 64.3 | 100-499 | 1 - 5% | <1% |
| 12 | Pain | High | <10 | 100.0 | 100-499 | 1 - 5% | 1 - 5% | | Low | <10 | 60.0 | <100 | <1% | <1% |
| 13 | PC | High | 10-24 | 90.0 | <100 | <1% | <1% | | High | <10 | 80.0 | <100 | <1% | <1% |
| | Pain | High | 10-24 | 100.0 | <100 | 1 - 5% | <1% | | High | <10 | 87.5 | <100 | <1% | <1% |
| 14 | MH | Low | 10-24 | 72.7 | 100-499 | 1 - 5% | 1 - 5% | | Medium | 10-24 | 75.0 | <100 | >5% | >5% |
| | PC | Low | 25-49 | 63.0 | 100-499 | <1% | <1% | | Medium | 25-49 | 61.3 | 100-499 | 1 - 5% | <1% |
| 15 | PC | High | 25-49 | 67.4 | 100-499 | 1 - 5% | <1% | | High | ≥50 | 73.8 | 100-499 | 1 - 5% | <1% |
| 16 | PC | Medium | ≥50 | 54.7 | 100-499 | >5% | >5% | | High | ≥50 | 61.3 | ≥500 | >5% | 1 - 5% |
| | PC | High | 10-24 | 100.0 | <100 | <1% | <1% | | High | 10-24 | 36.4 | <100 | <1% | <1% |
| | PC | High | 10-24 | 100.0 | <100 | <1% | <1% | | High | 10-24 | 25.0 | <100 | <1% | <1% |
| | Pain | High | 10-24 | 90.5 | 100-499 | <1% | <1% | | High | 10-24 | 60.0 | <100 | <1% | <1% |
| 17 | Pain | High | 10-24 | 70.0 | 100-499 | 1 - 5% | <1% | | High | <10 | 50.0 | 100-499 | <1% | <1% |
| | MH | High | ≥50 | 84.9 | 100-499 | <1% | <1% | | High | 25-49 | 77.1 | 100-499 | 1 - 5% | <1% |
| | Pain | High | <10 | 80.0 | 100-499 | <1% | <1% | | High | <10 | 62.5 | <100 | <1% | <1% |
| 18 | PC | High | 25-49 | 58.8 | 100-499 | <1% | <1% | | High | ≥50 | 90.6 | 100-499 | <1% | <1% |
| | PC | High | <10 | 75.0 | <100 | <1% | <1% | | High | 10-24 | 94.1 | <100 | <1% | <1% |

No. = Number
PC = Primary Care
MH = Mental Health
OUD = Opioid Use Disorder
MOUD = Medications for Opioid Use Disorders
a = Facility complexity is based on a VA constructed index that classifies VA facilities into three main categories: high complexity (level 1a-1c), medium complexity (level 2), and low complexity (level 3). Facility complexity is based on the number of patients, patient risk scores and cost of patient population, research funding, and availability of inpatient and outpatient clinical services.
b = Number of prescribers
c = Percent of physician prescribers among all prescribers
d = Number of patients with documented OUD
e = Percent of patients with OUD who received MOUD
f = Percent increase in MOUD receipt among patients with documented OUD

© 2021 Hawkins EJ et al. *JAMA Network Open.*

**eTable 2. Integrated Promoting Action on Research Implementation in Health Services Framework Elements and SCOUTT Initiative Interventions**

| Elements | Implementation Strategies | SCOUTT Interventions | Fidelity Monitoring |
|---|---|---|---|
| Facilitation | • External facilitation | • Each site assigned an external facilitator with regular meetings | Number of meetings per site (Mdn = 6, range = 0-10)<br>83.3% of sites with >2 meetings |
| Innovation | • Conduct educational meetings<br>• Conduct ongoing training<br>• Distribute educational materials<br>• Promote adaptability<br>• Provide ongoing consultation | • In-person two-day training<br>• Monthly webinars to address knowledge gaps<br>• SharePoint site with online training resources<br>• On-going consultation | 246 attendees representing medical, behavioral health, pharmacy and leaders<br>Data unavailable<br><br>Data unavailable |
| Recipients | • Identify and prepare clinical champions<br>• Create a learning community | • In-person two-day training<br>• Monthly meetings to promote learning community | See above<br>Number of attendees per meeting (M = 66.4, SD = 9.7). |
| Context | • Create new clinical teams<br>• Prioritize change<br>• Audit and provide feedback | • Formation of interdisciplinary teams<br>• National leadership requesting all regional networks involvement in SCOUTT initiative<br>• Quarterly reports | All sites developed implementation teams<br>Memorandum sent to all regional network directors<br>Four quarterly reports sent by email to all sites |

Mdn = median
M = mean
SD = standard deviation

© 2021 Hawkins EJ et al. *JAMA Network Open.*

**eTable 3. Sensitivity Analyses - Association Between the Implementation Intervention and Monthly Proportion of Patients Receiving MOUD Using Mixed Effects Logit Models with Site Only as Random Effect**

| | **Pre-implementation** | | **Post-implementation** | | **Step Change** | |
|---|---|---|---|---|---|---|
| | AOR (95% CI)[a] | P-value | AOR (95% CI)[a] | P-value | AOR (95% CI)[a] | P-value |
| Intervention clinics | 1.02 (1.01-1.04) | 0.003 | 1.05 (1.03-1.07) | <0.001 | 1.09 (0.97-1.22) | 0.141 |
| Comparison clinics | 1.03 (1.01-1.04) | 0.002 | 0.99 (0.97-1.02) | 0.586 | 0.84 (0.73-0.97) | 0.018 |
| Intervention vs. Comparison clinics | 1.00 (0.98-1.02) | 0.704 | 1.05 (1.02-1.08) | <0.001 | 1.27 (1.06-1.52) | 0.011 |

a = Adjusted for demographic (age, gender, race/ethnicity, marital status, housing insecurity, and VA disability rating $\geq$50%), mental health comorbidity (depression, anxiety, posttraumatic stress disorder, serious mental illness disorders[1]), substance use comorbidity (alcohol use and any non-opioid drug use disorders), medical comorbidity, clinic type (primary care, mental health, pain), and time-varying indicators (monthly visit attendance in intervention/comparison clinics, monthly substance use disorder specialty-care attendance, and monthly medications for opioid use disorder receipt from other VA and non-VA clinics).

AOR = Adjusted Odds Ratio

CI = Confidence Interval

1 = Serious Mental Illness Disorders include schizophrenia-spectrum or bipolar-spectrum disorders.

© 2021 Hawkins EJ et al. *JAMA Network Open.*

## eTable 4. Yearly Unadjusted Percentages of MOUD Receipt among Patients in Comparison and Intervention Clinics

| | Patients Receiving MOUD in Comparison clinics | | | | Patients Receiving MOUD in Intervention clinics | | | |
|---|---|---|---|---|---|---|---|---|
| | Pre-implementation Year | | Post-implementation Year | | Pre-implementation Year | | Post-implementation Year | |
| | No. | % (95% CI) | No. | % (95% CI) | No. | % (95% CI) | No | % (95% CI) |
| Any MOUD | 294 | 4.8 (2.7 – 8.6) | 390 | 6.4 (3.6 – 11.1) | 472 | 6.3 (3.1 – 12.4) | 1,016 | 13.6 (8.1 – 21.8) |
| Buprenorphine | 276 | 4.5 (2.4 – 8.2) | 372 | 6.1 (3.4 – 10.6) | 413 | 5.5 (2.5 – 11.6) | 944 | 12.6 (7.3 – 20.8) |
| Injectable Naltrexone | 18 | 0.3 (0.1 – 0.7) | 19 | 0.3 (0.1 – 0.9) | 63 | 0.8 (0.3 – 2.1) | 74 | 1.0 (0.5 – 2.0) |

MOUD = Medications for Opioid Use Disorder
No. = Number
CI = Confidence Interval

© 2021 Hawkins EJ et al. *JAMA Network Open.*
